# Challenging the selection for consistency in the rank of endurance competitions

**DOI:** 10.1186/s12711-020-00539-5

**Published:** 2020-04-10

**Authors:** Isabel Cervantes, Loys Bodin, Mercedes Valera, Antonio Molina, Juan Pablo Gutiérrez

**Affiliations:** 1 Departamento de Producción Animal, Universidad Complutense de Madrid, Avda. Puerta del Hierro s/n, 28040 Madrid, Spain; 2 GenPhySE, INRAE, 31320 Castanet-Tolosan, France; 3 Departamento de Ciencias Agro-Forestales, Universidad de Sevilla, Ctra. Utrera km 1, 41013 Seville, Spain; 4 Departamento de Genética, Universidad de Córdoba, Campus de Rabanales, 14071 Córdoba, Spain

**Keywords:** Genetic parameters, Horse, Canalization, Ranking

## Abstract

**Background:**

Control of the environmental variability by genetic selection offers possibilities for new selection objectives for productive traits. This methodology aims at reducing heterogeneity in productive traits and has been applied to several traits and species for which animal homogeneity is profitable. In horse breeding programmes, rank in competitions is a common selection objective but has been challenging to model. In this study, the parameters of environmental variability for the rank of a horse were computed to analyse the capability of a horse to maintain the best ranking across competitions that consist of long-distance races in which the adapted physical condition of the horse is essential. The genetic component of the environmental variance for the rank in endurance competitions was evaluated, which resulted in proposing a new transformation of horse scores in competitions.

**Results:**

Homogeneous and heterogeneous variance models were compared by assaying three random effects that affect both the rank and its variability, using endurance ride data consisting of 2863 records. The pedigree relationship matrix contained 5931 animals. The rank trait was transformed into a normalized variable to prevent false estimates of the genetic correlation by inappropriate artificial skewness. The models included the number of participants in the race, sex, and age as systematic effects. The rider, the rider-horse interaction, or an environmental permanent effect were tested as random effects, in addition to additive genetic and residual effects. The models were analysed using the GSEVM program. Estimates of heritability for rank ranged from 0.12 to 0.15. The heterogeneous variance model that fitted the rider was assessed as the best model based on the deviance information criterion. Estimates of genetic variance for rank variability ranged from 0.12 to 0.13. The genetic correlation between the rank and its environmental variability was low and did not differ from 0.

**Conclusions:**

These results offer an opportunity to select animals for canalization by reducing the variability of race results and achieving the best positions, which could be a new selection objective by weighting estimated breeding values for rank and its variability in a selection index.

## Background

The main goal of horse breeding programmes is to enable animals to reach preferential positions in a variety of competitions. Genetic evaluation applied to horse competitions is a challenge because the trait of interest is not recorded as a quantitative trait, but rather as the rank the animal reaches. Genetic evaluation of ranking in races is one of the most persistent issues in horse breeding [1, 2]. Apart from the discrete nature of the records, their distribution presents at least two issues: (1) they have a uniform distribution within a race if the rank is expressed over the number of participants, and (2) they have a right-skewed distribution across races due to the different numbers of participants if the rank is not standardized by participants. Several useful models or transformations are available to address these problems [3–5]. Among the methodologies, those that perform best apply the Thurstonian approaches, which are competitive models that describe the genetic ability of animals to be in the front places of a race as a continuous unobserved value [6]. These competitive models have been used for other sport performance events, such as those involving trotter horses [7], but were demonstrated to perform best in endurance races [8]. However, Thurstonian models do not apply to the genetic analysis of variability of rank, and appropriate transformations are needed for that purpose [9].

Endurance competitions are cross-country long-distance races that range from 40 to 160 km per day and combine speed, endurance, and physical condition of horses for the competition. The rank of horses ranges from first to the number of participants, regardless of their average level. A good position in a short race would not necessarily be ideal in a long race, but a breeder might be interested in breeding animals that are competitive for the best position regardless of the race conditions (e.g. length, altitude, type of surface). This results in a selection objective that combines a reduction of the variability of the result in a race with the best position being reached, with specific weights in a selection index. Selection for modifying the variability has been proven to be possible in several species, mainly in experimental studies on prolific and short-generation interval species, such as mice [10–12], rabbits [13–17], and even pigs [18], but it has not been studied in depth in horses. Another possible controversial issue is the correlated response in the mean of the trait when selecting for reduced variability, which depends on the genetic correlation between the trait and its environmental variability. A wide range of genetic correlations between mean and variability was reported by Hill and Mulder [19], from highly negative to highly positive. This genetic correlation has to be estimated at the same time as the genetic variance of the environmental variability. Transformation of the variability of the trait is recommended given its characteristics, but it is important to check that the transformation does not lead to skewed distributions that, in turn, would cause a spurious correlation from a mathematical artefact [20].

A concern about models that analyse data from horse competitions is the influence of the rider and of rider-horse interactions [21–23]. Rider-horse interactions, known as the ‘match’ effect, involves the relationship, communication, and cooperation between rider and horse, which is influenced by the level of experience and behaviour of both the rider and the horse [24]. However, in data registered during horse competitions, the inclusion of several additional random effects in the model in addition to the genetic effect has been studied, but the optimal set of effects to be included remains unclear [8, 25].

The objective of this study was to estimate the genetic parameters that are relevant to the variability of the position of horses in endurance races, including the genetic correlation between the variability of position and the position by defining an appropriate transformation of the trait. The effects of including the rider, the rider-horse interaction, or permanent environmental effects as additional random effects in the model were also evaluated.

## Methods

The total dataset consisted of 2863 ranking records from 621 horses (238 males, 253 females and 130 geldings) aged between 5 and 24 years, with at least two records per horse. Most of the horses were Arabian horses (69.6%), and the remaining were Anglo-Arabian horses (20.5%), Spanish sport horses (5.5%), and horses of other breeds (4.4%). The records were collected during 581 endurance races held in Spain (92% of records), in France (6%), and in other countries (2%), between 2000 and 2016. Records on non-placed animals were removed from the dataset. The number of records per horse ranged from 2 to 22, with an average of 4.6 records per horse.

The number of different riders in the dataset was 612. The average number of riders who rode a particular horse was 1.9; 56.8% of horses were ridden by different riders. The average number of horses that were ridden by a given rider was 1.95; 40.8% of riders rode at least two horses. The rider-horse interaction (match effect) was based on the rider-horse pair and evaluated differences in the horse’s behaviour with specific riders. The match effect had 1096 levels, with on average 2.4 records per level.

The pedigree information for genetic evaluation totalled 5931 animals. The mean number of equivalent complete generations was 6.9, as computed with the ENDOG4.8 software [26].

**Fig. 1:**
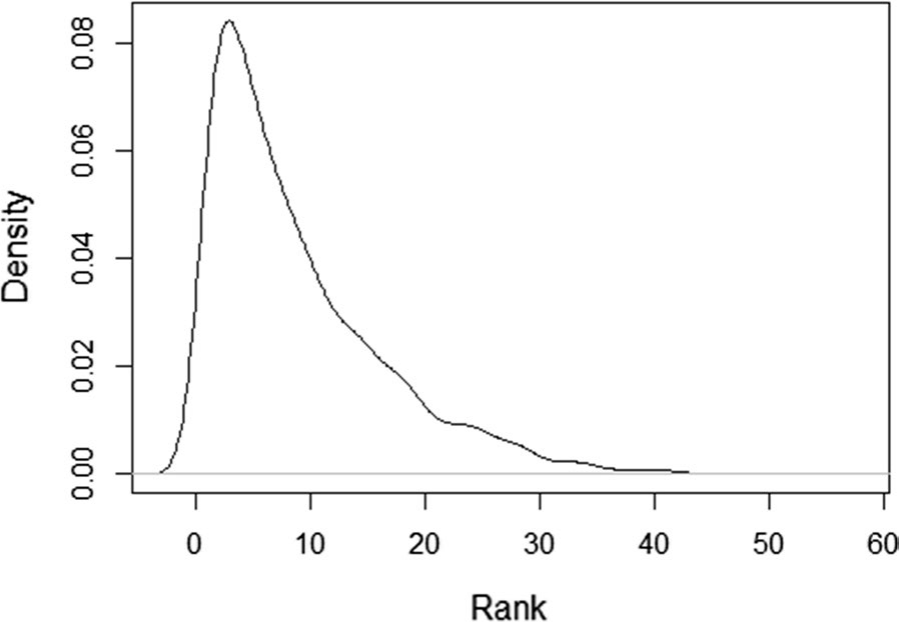
Distribution of rank positions based on the original data

**Fig. 2:**
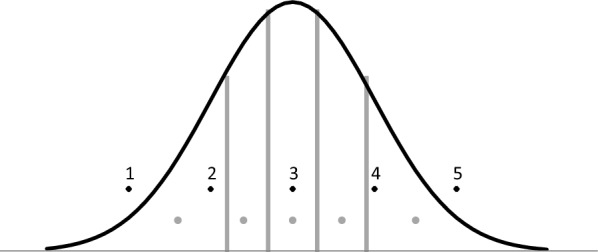
Example of the transformation performed for a race with five participants. The black circles indicate the uniform distribution of original ranks, the bars are the created thresholds, and the grey circles are the new values in the underlying normal distribution

**Fig. 3:**
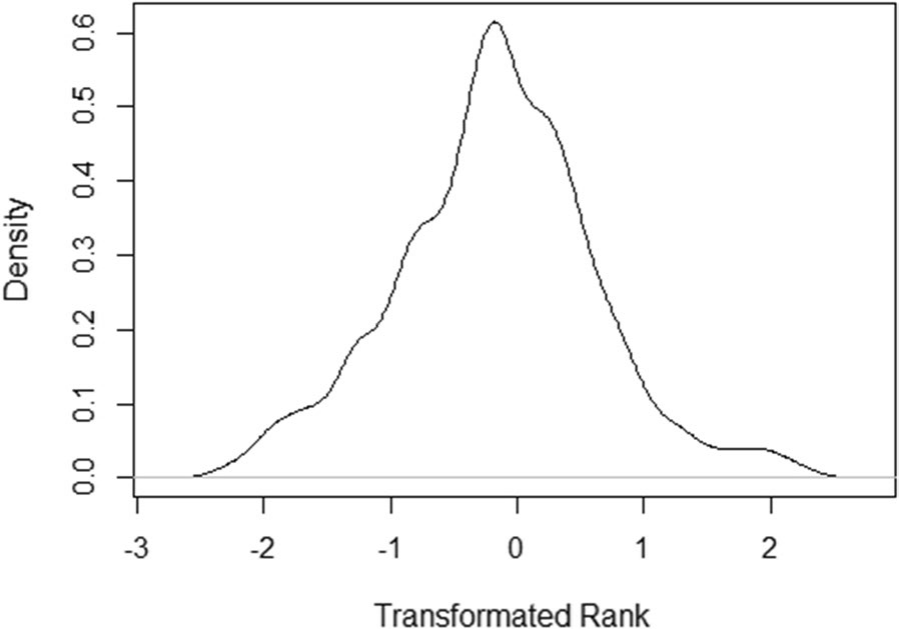
Distribution of ranks after transformation

The distribution of the original rank records is given in Fig. 1, which shows a right skewness because, unlike high positions, first positions are always defined in races, even those with a small number of participants. The average number of records per event was 4.9, with a minimum of 2 and a maximum of 16. A transformation was indicated because the skewness can artificially determine the sign of the genetic correlation between the trait and its variability [10, 20]. In some events, only the rank of some of the horses was registered, and the recorded ranks were not consecutive. In addition, the number of participants was frequently missing and had to be estimated. We estimated the number of participants in a race as the maximum of the average position multiplied by 2, rounded to the next integer, and the maximum rank registered for the race in the data. Since this way of scoring led to a uniform distribution of the scores, a transformation was performed by splitting a standard normal distribution in N equiprobable bins, where N is in the number participants in the race, and computing the expected ordinate value of each bin using the inverse normal function. Figure 2 shows an example of how the transformation was performed for a race with five participants. Note that the transformed variable places each record on an underlying scale. For example, a horse will be first in a race of two participants if the transformed variable was lower than 0, first in a race of three if its value was lower than − 0.599, first in a race of four if its value was lower than − 0.967, first in a race of five if its value was lower than − 1.175, and so on. These values were the thresholds on the left side of the distribution, with a density of 1/2, 1/3, 1/4, and 1/5. The final distribution of the transformed variable was checked to confirm that it had a normal distribution, but the result had very low skewness (Fig. 3). As a result, the distribution of the trait is not expected to have a large influence on the genetic correlation between the trait and its variability.

Models assuming homogeneity and heterogeneity of the residual variance were fitted to the transformed trait ($y_{i}$) as follows:

Homogeneity model (HO):

$$y_{i} = {\mathbf{x}}_{i} {\mathbf{b}} + {\mathbf{z}}_{i} {\mathbf{u}} + {\mathbf{w}}_{i} {\mathbf{r}} + e_{i} .$$

Heterogeneity model (HE):

$$y_{i} = {\mathbf{x}}_{i} {\mathbf{b}} + {\mathbf{z}}_{i} {\mathbf{u}} + {\mathbf{w}}_{i} {\mathbf{r}} + e^{{\frac{1}{2}\left( {{\mathbf{x}}_{i} {\mathbf{b}}^{*} + {\mathbf{z}}_{i} {\mathbf{u}}^{*} + {\mathbf{w}}_{i} {\mathbf{r}}^{*} } \right)}} \varepsilon_{i} .$$

where * indicates the parameters associated with residual variance, ${\mathbf{b}}$ and ${\mathbf{b}}^{*}$ are vectors of the systematic effects, ${\mathbf{u}}$ and ${\mathbf{u}}^{*}$ are vectors of the additive genetic effects, ${\mathbf{r}}$ and ${\mathbf{r}}^{*}$ vectors of an additional random effect, and ${\mathbf{x}}_{i}$, ${\mathbf{z}}_{i}$, and ${\mathbf{w}}_{i}$ are incidence vectors for systematic, animal, and additional random effects, respectively. Finally, $\varepsilon_{i} \sim N\left( {0, 1} \right)$. The genetic effects ${\mathbf{u}}$ and ${\mathbf{u}}^{*}$ were assumed to be Gaussian:

$$\left[ {\begin{array}{*{20}c} {\mathbf{u}} \\ {{\mathbf{u}}^{*} } \\ \end{array} } \right]\sim N\left( {\left[ {\begin{array}{*{20}c} {\mathbf{0}} \\ {\mathbf{0}} \\ \end{array} } \right], \left[ {\begin{array}{*{20}c} {\sigma_{u}^{2} } & {\rho \sigma_{u} \sigma_{{u^{*} }} } \\ {\rho \sigma_{u} \sigma_{{u^{*} }} } & {\sigma_{{u^{*} }}^{2} } \\ \end{array} } \right] \otimes {\mathbf{A}}} \right),$$

where ${\mathbf{A}}$ is the additive genetic relationship matrix, $\sigma_{u}^{2}$ is the additive genetic variance of the trait, $\sigma_{{u^{*} }}^{2}$ is the additive genetic variance of residual variance of the trait, $\rho$ is the genetic correlation between the trait and its residual variability, and $\otimes$ denotes the Kronecker product. Vectors ${\mathbf{r}}$ and ${\mathbf{r}}^{*}$ were also assumed to be independent, with ${\mathbf{r}} \sim N\left( {0,{\mathbf{I}}_{{\mathbf{r}}} \sigma_{r}^{2} } \right)$ and ${\mathbf{r}}^{\varvec{*}} \sim N\left( {0,{\mathbf{I}}_{{\mathbf{r}}} \sigma_{{r^{*} }}^{2} } \right)$, where ${\mathbf{I}}_{{\mathbf{r}}}$ is the identity matrix of equal order to the number of levels of the corresponding random effect, and $\sigma_{r}^{2}$, and $\sigma_{{r^{*} }}^{2}$ are variances for the additional random effects associated with the mean and residual variability, respectively, of the trait [27]. The model applied included the effects of sex (male, female, gelding), number of participants in the race (covariate) in ${\mathbf{b}}$ and ${\mathbf{b}}^{*}$, and age at the time of competition, with ten levels: 5 to 6 years (162 records), one-year classes for horses from 7 to 14 years old (101 to 519 records), and horses more than 14 years old (86 records).

Within the HO and HE models, three models were assayed according to the nature of this additional random effect:

- Model R: Rider effect with 612 levels.
- Model RH: Rider-horse match effect with 1196 levels.
- Model P: Permanent environmental effect, a second horse effect different from the genetic effect, with 621 levels.

Variance components were estimated using the Bayesian procedure of the GSEVM software [28]. For comparative purposes, since residual variance is not unique in the HE models, a residual variance can also be estimated for a particular level of systematic effects. A global residual variance was estimated in the HE models by adding the averages of the estimates of all the levels within systematic effects. To maintain estimability of the corresponding linear combination, solutions for all levels of each of the other systematic effects were averaged within all the remaining systematic effects and added to the solution for that particular desired level of the systematic effect. Then, the global heritability (h^2^) for the position and for each level of systematic effect was computed [29]. The deviance information criterion (DIC) [30] was used to evaluate and compare the overall adequacy of the models.

## Results

Table 1 shows the estimates of variance components and genetic parameters obtained from the six models, as well as the DIC value for each model as a comparative measure of fitness. Based on the DIC criterion, any HE model was preferable to any HO model. For both the HO and HE models, inclusion of rider as an additional random effect produced the best fit, while inclusion of a permanent random effect resulted in the worst fit. Heritability of rank ranged from 0.12 (HE-P) to 0.15 (HO-RH), and given their standard deviation, we considered that there was no difference between these values. HE models tended to give slightly higher estimates of the global residual variance because they express the variance in an intermediate scenario within all systematic effects. The HE models assume that records are balanced across the dataset, whereas in the real dataset they are unbalanced. This resulted in lower heritability estimates for rank based on the HE models, but this was only an artefact due to unbalanced data.

**Table 1:** Estimates of variance components and parameters for rank and its variability for the homogeneous (HO) and heterogeneous (HE) models with additional random variables of rider (R), rider by horse (RH), and permanent environment (P)

Model	HO-R	HO-RH	HO-P	HE-R	HE-RH	HO-P
***Genetic parameters for score***						
Additive	0.090 (0.064, 0.116)	0.096 (0.069, 0.126)	0.082 (0.055, 0.111)	0.089 (0.064, 0.116)	0.096 (0.068, 0.124)	0.081 (0.055, 0.110)
Additional	0.102 (0.073, 0.131)	0.093 (0.065, 0.122)	0.077 (0.052, 0.105)	0.098 (0.072, 0.126)	0.087 (0.059, 0.113)	0.078 (0.052, 0.105)
Residual^a^	0.453 (0.426, 0.480)	0.448 (0.420, 0.476)	0.482 (0.455, 0.510)	0.483 (0.401, 0.563)	0.472 (0.397, 0.552)	0.509 (0.430, 0.595)
Phenotypic	0.644 (0.606, 0.683)	0.637 (0.600, 0.673)	0.644 (0.604, 0.679)	0.669 (0.586, 0.754)	0.654 (0.572, 0.734)	0.668 (0.587, 0.759
Heritability	0.139 (0.102, 0.177)	0.151 (0.112, 0.194)	0.128 (0.088, 0.170)	0.133 (0.095, 0.173)	0.146 (0.106, 0.191)	0.122 (0.081, 0.164)
***Genetic parameters for variability***						
Additive				0.117 (0.066, 0.174)	0.130 (0.072, 0.195)	0.121 (0.068, 0.182)
Additional				0.144 (0.077, 0.210)	0.143 (0.071, 0.215)	0.116 (0.065, 0.173)
cov(u,u*)				− 0.016 (− 0.052, 0.023)	− 0.016 (− 0.052, 0.023)	− 0.013 (− 0.053, 0.030)
r(u,u*)				− 0.157 (− 0.505, 0.244)	− 0.116 (− 0.487, 0.258)	− 0.133 (− 0.548, 0.304)
DIC	1023	1053	1121	863	895	1006

High posterior density intervals are in brackets (HPD95) of their marginal posterior distribution. DIC: Deviance information criterion

^a^in HE models, this is a global residual variance in an averaged scenario of systematic effects

Estimates of the genetic variance for the variability of rank ranged from 0.12 (HE-R) to 0.13 (HE-RH). The estimate of the correlation between the genetic variance of rank and its environmental variability was low and negative, ranging from − 0.12 (HE-RH) to − 0.16 (HE-R), but did not differ from 0.

The HE-R model had the lowest DIC and was assumed to be the best. Thus, in the rest of the paper, we will only discuss results from this model.

**Fig. 4:**
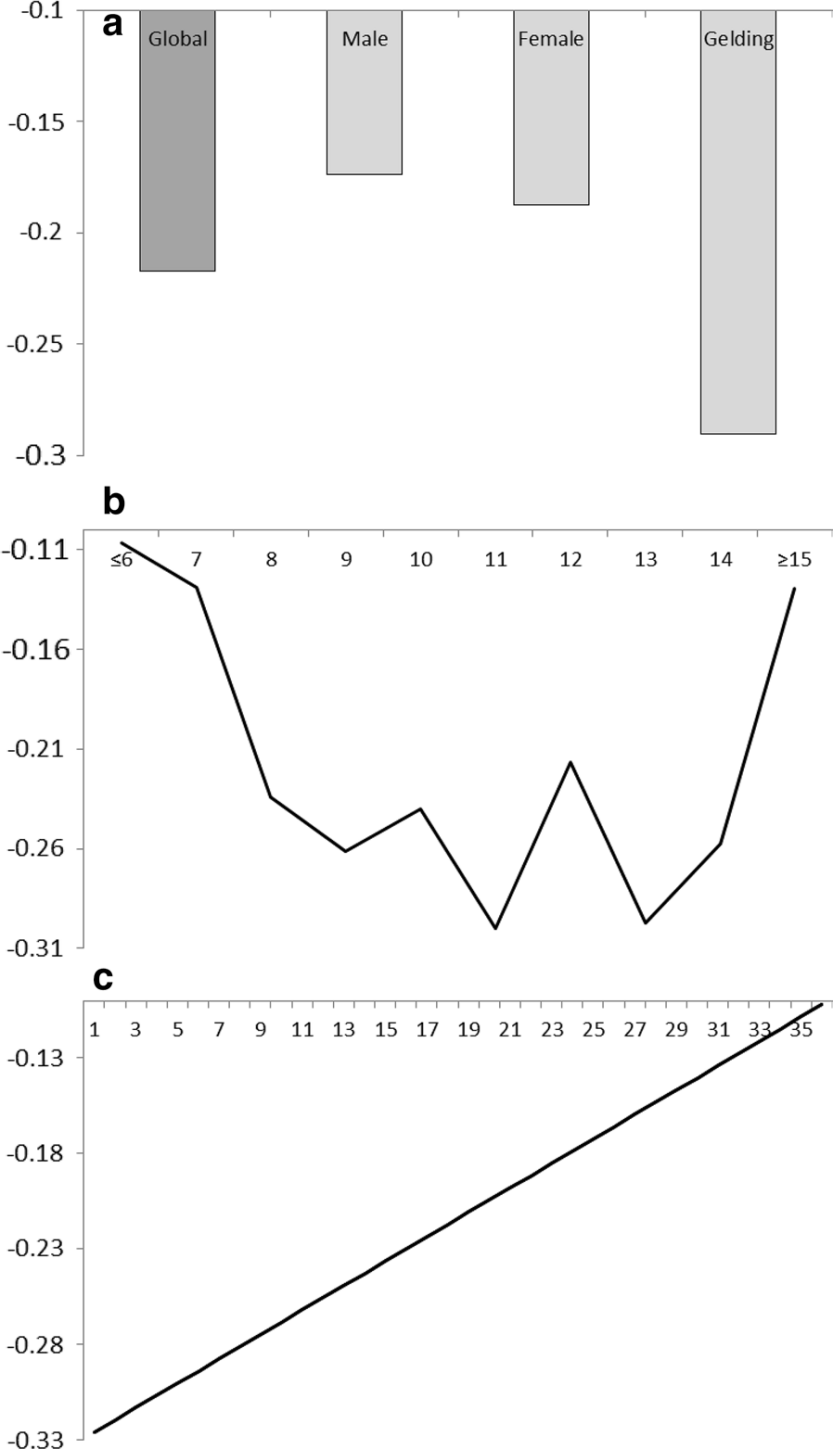
Mean scores on the transformed scale by the level of systematic effects: **a** sex, **b** age, and **c** number of participants

Phenotypic values that were computed using the transformed variable were also analysed. Figure 4 shows the mean values on the transformed scale according to the systematic effects: (a) mean score and sex, (b) age, and (c) number of participants. All the estimated means in this population were negative on the transformed scale, which shows that a horse that scored in an good position had a higher probability of being registered than another horse that scored in a poor position, as demonstrated by the slightly higher density of the right half of the distribution of the transformed score (Fig. 3). Geldings tended to achieve better positions, with non-castrated males tending to achieve a worse position (Fig. 4a). As expected, horses of intermediate ages tended to perform better (Fig. 4b), although the average performance of these horses was worse as the number of participants increased (Fig. 4c).

**Fig. 5:**
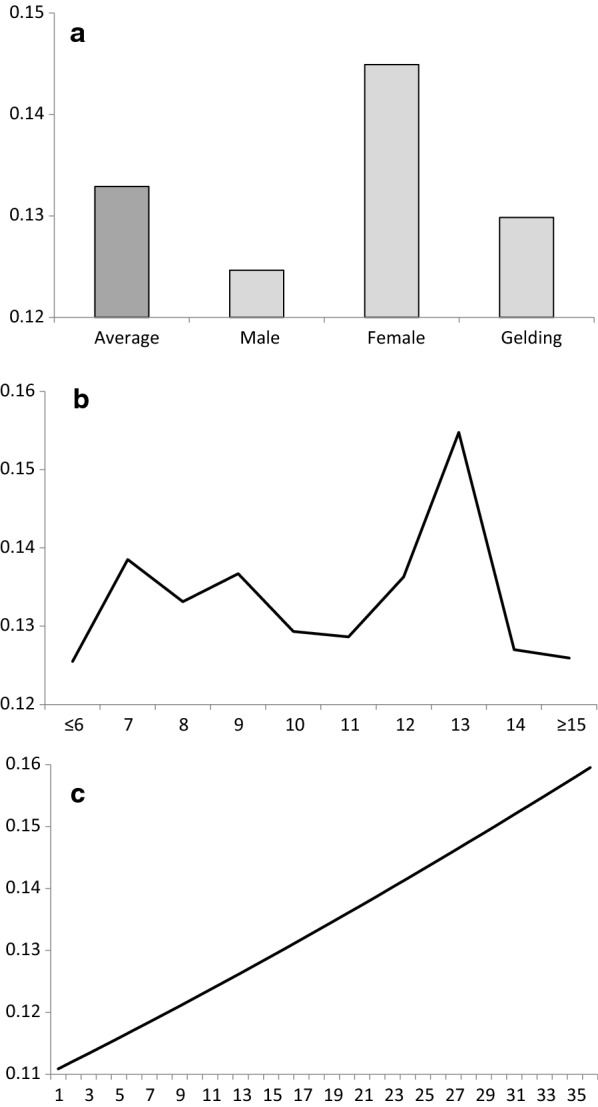
Heritability estimates for score on the transformed scale by the level of systematic effects: **a** average scenario and sex, **b** age, and **c** number of participants

Figure 5 shows the estimates of heritability for score on the transformed scale according to the systematic effects. The estimate of heritability for the average scenario of fixed effects was 0.13, with females being less variable in performance and having a small increase in heritability of up to 0.15, while males were more variable, with a heritability as low as 0.13 (Fig. 5a). The residual variance showed a tendency to increase with age of the animals, but there was an inexplicable dramatic decrease for the 13-year-old group (Fig. 5b), this group showing a tendency to score similar positions. In addition, as expected, an increase in the number of participants helped the animals to score similar positions across races (Fig. 5c) but also to obtain worse positions (Fig. 4c).

## Discussion

Genetic evaluation using data obtained from horse competitions is a difficult task, since the data consist only of positions in competitions and do not directly express equivalent differences in a hypothetical underlying variable that expresses performance abilities. Another challenge for genetic evaluation is the interest to select for reduced environmental variability, with the typical aim that breeding animals not only have an optimal performance but also maintain their performance across races. Finally, a third important issue in genetic evaluation of horses is to define the most appropriate model, particularly which random effects should be included [8, 25, 31]. In this paper, we address these three topics together by proposing a new transformation of horse competition scores. We compared homogeneity and heterogeneity models and evaluated three random effects that affect both the score and its variability.

Regarding the transformation used, the strong right skewness observed in the original distribution (Fig. 1) was completely removed by the transformed variable (Fig. 3). Nevertheless, a slightly larger number of records was found on the left side of the distribution than on the right side, which suggests a trend to preferably register animals that finish in good positions, rather than those placed in worse positions. The transformation also resulted in the mean of the distribution to be negative (− 0.16), and the least-squared global mean value to be − 0.22 (Fig. 4), even when the assumed balance was not skewed and the distribution was theoretically centred at zero (Fig. 2). This slight skewness might be partially influenced by how the number of participants was estimated for each race. Nevertheless, the genetic correlation estimated between the score and its environmental variability was low for all the models (Table 1), even when the negative estimates reflect a slight skewness observed on the left of the distributions.

Regarding additional random effects to include in the model, the worst-fitting model was the model that included the permanent effect (HO-P or HE-P), thus confirming the results by García-Ballesteros et al. [8], who used Thurstonian, threshold, and linear models in the same population. However, and unlike the results by García-Ballesteros et al. [8], the model that fitted the rider (HO-R or HE-R) performed better than the model that fitted the interaction (HO-RH or HE-RH).

The HE models obtained a better fit than any of the HO models, which confirmed that heterogeneity models tend to give preferable results than classical homogeneity models [10, 31]. García-Ballesteros et al. [8] showed that Thurstonian models were far better than the threshold and linear models by accommodating the idiosyncrasies of horse competition data. Thurstonian models can define different distances among consecutive participants in the underlying working variable. Unfortunately, extension of Thurstonian models to genetic heterogeneity models has not yet been developed.

Regarding systematic effects, geldings performed better (Fig. 4a), but females showed less residual variance and were the most stable (Fig. 5a), while males performed worse for both rank and variability. These results show the effect of castrating males for better performance, but then these animals cannot pass their genetic value to the next generations. Some other considerations have to be carefully studied. The influence of age was not clearly assessed, although horses with intermediate ages seemed to achieve the best positions. The animals tended to decrease in homogeneity with age, but inexplicably, not for horses between 12 and 13 years of age. Finally, performance decreased and consistency of placing increased with an increase in the number of participants. It is evident that animals that do not have the ability to achieve a good score in a specific race, do not strive much.

Another issue is the use of the predicted breeding values for variability to select animals to improve their consistency. This trait is particularly difficult to manage across generations since no phenotypic trend regarding positions or variability exists. Taking the population as a whole, selection for better performance leads to genetic improvement but not to phenotypic improvement, because races will include horses that perform better than others. Using predicted breeding values for variability of selection could create difficulties since the objective is to reduce this variability for horses that tend to win, whereas it should be high for horses that do not have a tendency to win. The best horse for bringing higher earnings would be a horse with consistent performance. For low-performance animals, it is preferable that they have heterogeneity characteristics so that they can sometimes win a race, but this quality is not desired by breeders.

The additional random effects are sometimes difficult to predict because of limited data within levels, for example, riders riding more than one horse [8, 24, 25]. The correlation between the predicted breeding values for environmental variability and the rider effect solution was 0.42, which means that a horse with variable performance was linked to a rider who also had variable performance.

We showed that selecting for good score performance and for consistent position can be achieved independently, since the genetic correlation between the score and its variability was low and did not differ from 0. The correlation between predicted breeding values for rank using the heterogeneous and homogenous models was almost 1, which shows the usefulness of the proposed transformation. Breeders can opt to increase the probability of winning or the security of a particular result. Both of these objectives can be combined into a selection index, with the consequent reduction of expected responses in both when the selective effort is distributed.

## Conclusions

We demonstrate the possibility of selecting simultaneously for improved ability to win and consistent performance across races. The transformation of scores into a variable that can accommodate a Gaussian distribution resulted in an alternative approach for dealing with the rank trait. In contrast to the original rank, with this transformed variable, models that accounted for heterogeneity of residual variance were preferable, and fitting the rider as an additional random effect attained the best results in terms of DIC. The estimate of the genetic correlation between rank and its variability was low and did not differ from 0, thus leading to the possibility to simultaneously select for the ability to win and perform with consistency.

## Data Availability

The datasets generated and/or analysed during the current study are available from the corresponding author upon reasonable request.
